# Addressing the Needs of the Rapidly Aging Society through the Development of Multifunctional Bioactive Coatings for Orthopedic Applications

**DOI:** 10.3390/ijms23052786

**Published:** 2022-03-03

**Authors:** Tinkara Mastnak, Uroš Maver, Matjaž Finšgar

**Affiliations:** 1 Laboratory for Analytical Chemistry and Industrial Analysis, Faculty of Chemistry and Chemical Engineering, University of Maribor, Smetanova ulica 17, 2000 Maribor, Slovenia; tinkara.mastnak@um.si; 2 Institute of Biomedical Sciences, Faculty of Medicine, University of Maribor, Taborska ulica 8, 2000 Maribor, Slovenia; uros.maver@um.si; 3 Department of Pharmacology, Faculty of Medicine, University of Maribor, Taborska ulica 8, 2000 Maribor, Slovenia

**Keywords:** biomaterials, orthopedic implants, multifunctional coatings, drug delivery, antimicrobial, pain relief

## Abstract

The unprecedented aging of the world’s population will boost the need for orthopedic implants and expose their current limitations to a greater extent due to the medical complexity of elderly patients and longer indwelling times of the implanted materials. Biocompatible metals with multifunctional bioactive coatings promise to provide the means for the controlled and tailorable release of different medications for patient-specific treatment while prolonging the material’s lifespan and thus improving the surgical outcome. The objective of this work is to provide a review of several groups of biocompatible materials that might be utilized as constituents for the development of multifunctional bioactive coatings on metal materials with a focus on antimicrobial, pain-relieving, and anticoagulant properties. Moreover, the review presents a summary of medications used in clinical settings, the disadvantages of the commercially available products, and insight into the latest development strategies. For a more successful translation of such research into clinical practice, extensive knowledge of the chemical interactions between the components and a detailed understanding of the properties and mechanisms of biological matter are required. Moreover, the cost-efficiency of the surface treatment should be considered in the development process.

## 1. Introduction

Science and technology have brought changes to our society not seen since the industrial revolution. The world’s population reached 7.7 billion in mid-2019, having added one billion people since 2007. In 2018, for the first time in human history, persons aged 65 years or over outnumbered children under five years of age worldwide. Between 2019 and 2050, the number of people aged 65 or over is projected to more than double [1].

Profound advancements nearly eradicated many fatal illnesses but caused the occurrence of another global threat: obesity. Obesity and being overweight have now become the norm, affecting 52% of the world’s population [2], and it is deemed to be a crucial cause of the rise in chronic conditions that are estimated to be responsible for 60% of deaths in the world [3]. Nowadays, chronic diseases represent the greatest universal health problem and are replacing short-term remedies with long-term management strategies [4].

The unprecedented aging of the world’s population will boost the need for orthopedic implants and expose their current limitations to a greater extent due to the medical complexity of elderly patients [5] and the longer indwelling times of implanted materials [6]. It is therefore essential to develop techniques that will prolong the lifespan of the materials used and improve surgical outcomes [7]. In order to reach these goals in an intricate biological system such as the human body, implants with multiple functions are necessary [8]. A class of materials that seems very promising as regards fulfilling these needs is biocompatible metals with multifunctional bioactive coatings [9,10,11,12]. Multifunctional bioactive coatings might improve the properties of the implanted biocompatible metal materials by enhancing their resistance to corrosion [13], enabling better integration with the bone tissue [14] while preventing the occurrence of device-related infections [15,16]. Moreover, bioactive coatings have been shown to be an efficient means for the controlled release of several types of active ingredients in vitro and in vivo [17,18,19,20].

For successful translation from in vitro studies to animal and clinical trials, scientific efforts should be directed towards discovering efficient methods of releasing clinically relevant pharmaceuticals and approved therapeutics rather than the discovery of novel molecules [21,22]. Therefore, the objective of this work is to provide a review of several groups of biocompatible materials that can be utilized as constituents in the development of multifunctional bioactive coatings on metal substrates, with a focus on antimicrobial, pain-relieving, and anticoagulant properties. Furthermore, this review presents an in-depth economic and medical rationale for the development of such materials while providing a summary of medications used in clinical settings. It additionally presents a brief review of biopolymers and inorganic biomaterials for the preparation of coatings that serve as carrier matrixes for the selected pharmaceuticals and govern their release kinetics. Lastly, the review points out the disadvantages of the commercially available products and provides insight into the latest development strategies.

## 2. Statistics on the Number of Orthopedic Procedures and the Rationale for Developing Enhanced

The global medical implant market is foreseen to reach a staggering USD 136.59 billion by 2023 [23], creating an extremely high demand for high-performance, implantable biomaterials. Among the segments thereof, orthopedic implants command the largest share of the market.

In 2019, North America represented the largest implantable biomaterials market, followed by Europe and Asia Pacific. As reported by the OECD in 2016, 1.5 million joint arthroplasties are performed in Europe annually [24], while the data for the United States show that, in 2015, around 7 million Americans (more than 2150 per 100,000 inhabitants) were living with a hip or knee replacement [25]. In the United States, total hip arthroplasty (THA) is one of the most rapidly growing procedures, with a projected increase of 174% in the next decade [26]. Implantable orthopedic metal materials are also used in osteosynthesis procedures (e.g., the operation of uniting the ends of a broken bone by a wire or metal plate), regarding which databases are much scarcer. However, an analysis performed in 2015 in France revealed that approximately 403 new osteosynthesis implants per 100,000 people were implanted [27], which is comparable to the number of joint arthroplasties performed (Table 1). The data provided by Eurostat for the year 2018 showed that Germany, Austria, Belgium, and Finland were the EU Member States with the highest frequencies of hip replacement. Furthermore, in EU member states with the highest number of knee replacements per 100,000 inhabitants (more than 200 in Finland, Austria, Germany, and Belgium), the frequencies were four times greater compared to countries such as Ireland, Bulgaria, and Romania, where fewer than 50 total knee replacements per 100,000 inhabitants were performed.

There is no doubt that the application of implants in orthopedic surgery was an advance that greatly improved the quality of people’s lives. Still, even an ideal metal material cannot completely fulfill the characteristics of human tissues and can only be a compromise in response to mechanical strains, physicochemical degradation, aging, wear, and tear [29].

Primary total hip and total knee arthroplasties are the most frequently performed orthopedic surgeries, and statistics show that infection rates are increasing with the number of performed procedures [30]. According to the literature, 10% of patients who undergo total hip arthroplasty (THA) may require revision surgery within the first 15 years of service, while some sources estimate that aseptic loosening, accompanied by periprosthetic osteolysis, accounts for at least 50% of all THA revisions [31].

Table 2 presents some data on hospital costs due to revision surgery after hip or knee prosthetic joint infection in Europe and the United States [32,33,34,35,36,37,38,39,40], in order to provide some actual numbers.

The costs presented in Table 2 vary between countries because different methodologies were used to calculate them. Moreover, there are significantly different infection management strategies among countries and even hospitals within the same country. The data on the cost of orthopedic-implant-induced infection treatments typically depend on the associated diseases, the type of treatment, and the antibiotic susceptibility profile. A well-known risk factor for infection is previous surgery [41]. The reported economic impacts usually consist of direct in-hospital costs, direct outpatient costs, and indirect costs (e.g., the loss of productivity and work absenteeism), which are practically impossible to determine [42]. Estimates of the incidence of prosthetic joint infection after primary knee replacement range between 0.85% in Germany, 1.0% in the UK, 1.4% in Finland, and 2.2% in the USA [41], whereas the risk of infection in primary THA is generally estimated to be less than 1% [35]. Klouche et al. published a cost-analysis approach to total hip arthroplasty revision due to infection [35]. Their data showed that the cost of treating an infected hip arthroplasty is 2.6 times greater than the cost of an aseptic revision and 3.6 times greater than the primary THA, thereby providing further evidence of the importance of the development of orthopedic implant materials that prevent the occurrence of infections.

Increased life expectancy inevitably leads to an increased likelihood of osteoporosis (loss of bone density), which puts the elderly at an even greater risk of having to experience a (complicated) orthopedic procedure. Moreover, polypharmacy, the simultaneous use of several medications, is increasingly common in this segment of the population [5] and is estimated to affect between 40% and 50% of older adults in high-income countries [43]. Polypharmacy is considered to be one of the most relevant age-related factors that contribute to a higher prevalence of adverse drug reactions, thus increasing morbidity, mortality, and healthcare costs [44]. Due to such medical complexity of elderly patients, it would be highly beneficial to enhance the performance of the currently used orthopedic implants, not only by prolonging their lifespan but also by enabling the controlled and tailorable release of different medications and therapeutic agents that improve surgical outcomes. Three-dimensionally printed biocompatible metals with multifunctional bioactive coatings are one of the materials currently considered to be quite promising for this purpose. The final product in the development of such materials needs to provide several effects at once.

First is the prevention of infection. Orthopedic implant-related infections are most frequently caused by *Staphylococci* species [12,45] and can trigger implant loosening, implant removal or detachment, amputation, and delayed wound healing.

Second is pain management. Pain is the most common symptom of infections caused by orthopedic implant failure. Apart from severe pain, frequent symptoms due to acute infection are swelling, erythema, and fever. On the other hand, chronic infection generally manifests as pain alone and is often accompanied by the loosening of the implant [30].

Third is the prevention of osteolysis. Osteolysis is often the result of chronic inflammation. Ongoing inflammation undermines the stability of the prosthesis and its ability to withstand physiological loads. Once osteolysis can be seen radiographically, it will inevitably progress, resulting in surgery that will correct the failing articulation and address prior and ongoing potential bone loss [31].

Fourth is enhanced corrosion resistance. The corrosion of orthopedic implants remains a partially unsolved challenge. The surface modification of metals can improve their biocompatibility and hence slow down the corrosion process after prolonged exposure to the aggressive conditions present in the human body.

Fifth is improved osseointegration. The selection of scaffold materials and their architectural design play a critical and increasingly complex role in promoting bone regeneration by providing mimicry of the native bone matrix [46]. By modifying specific surface characteristics on the implant, a more favorable interaction can be induced between the implant and the native bone, thus augmenting implant osseointegration and leading to its firm anchorage.

## 3. Biocompatible Metal Materials

**Figure 1:**
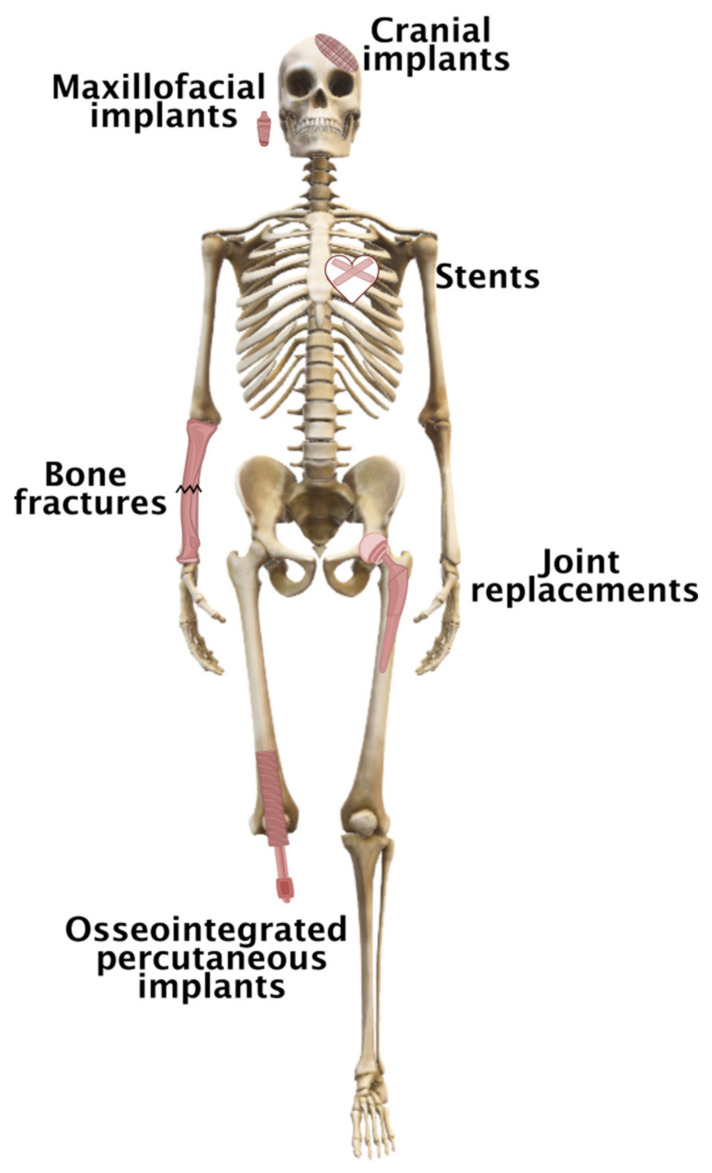
Some examples of metal-based implants used in the human body.

Implant components based on metallic elements (Figure 1) are used as biomedical materials because they have numerous advantages over other materials, including high mechanical strength, durability, good thermal and electrical conductivity, ductility, and chemical and biological compatibility. The most common metals used in implants are stainless steel, cobalt-based alloys, and titanium-based alloys.

Titanium and its alloys have been successfully employed as artificial implants in orthopedic surgery for decades. They are used especially in bone fusion, bone fixation, and joint arthroplasty [47]. The alloy Ti6Al4V (chemical composition 90% titanium, 6% aluminum, and 4% vanadium) has been extensively used in orthopedics due to its low density, high corrosion resistance, and excellent mechanical properties. Nonetheless, research suggests that vanadium and aluminum may have undesirable effects by increasing the expression of pro-inflammatory factors that cause osteolysis [48].

The desire to improve titanium alloys led to the development of several types of TiNb alloys without toxic and allergic alloying elements [49,50,51].

316L grade stainless steel is inexpensive, biocompatible, and possesses excellent mechanical and tribological properties [52], making it suitable for various applications. Due to its high availability and low cost, stainless steel has been used as an implant material for several decades. 316L grade stainless steel contains a high weight percentage of nickel, which can cause an allergic reaction in patients with recognized sensitivity to this metal [53]. Stainless steel metal implants are nowadays mostly used as materials for stems in total hip replacements and devices for bone fixation, such as nails, screws, and fracture plates [54].

Cobalt–chromium (CoCr) alloys are metallic materials often selected to manufacture implants used in heavy-loaded joints due to their excellent wear and corrosion resistance. According to the American Standards for Testing and Materials (ASTM), there are four types of CoCr alloys recommended for surgical implant applications, namely CoCrMo alloy (F75), CoCrWNi alloy (F90), CoNiCrMo alloy (F562), and CoNiCrMoWFe alloy (F563) [55]. Due to the release of Co, Cr, and Ni ions after longer periods of time, their presence in permanent implants is nowadays limited [56].

Another group of metal materials used in orthopedics is nickel–titanium (NiTi) shape memory alloys (SMAs). The shape memory effect means that they can return to their original shape after mechanical deformation if heated. The most known representative of NiTi SMAs is nitinol, an alloy containing a nearly equiatomic composition of titanium and nickel. Their shape memory effect, pseudoplasticity, and excellent biocompatibility are the reasons for NiTi SMAs being used as staples for foot or hand surgery [57]. The downside of NiTi SMAs is their challenging manufacture, which is reflected in their limited application [58].

Tantalum (Ta) is ductile, hard, corrosion resistant, and biocompatible and has been in clinical use since the mid-20th century [59]. The development of porous tantalum renewed research interest in this material. Porous tantalum has a high-volume porosity with fully interconnected pores that show potential for secure and rapid bone ingrowth [14]. Clinically used porous tantalum mainly refers to Trabecular Metal™, produced by Zimmer (Minneapolis, MN, USA) [60]. Ta has found application in hip and knee arthroplasty and spinal surgery [61], although the commercial success of Ta-based orthopedic implants has been hindered by their relatively high manufacturing cost.

Table 3 presents an overview of the metallic materials used in orthopedic devices cleared or approved by the FDA. Some of the commercially present metal materials used to manufacture orthopedic implants are also listed.

As seen in Table 3, several metal materials have been cleared or approved by the FDA for use in orthopedics, either as bone fixation devices, prostheses, or soft tissue fixation devices, but in practice, more than two thirds of orthopedic implants are fabricated from Ti alloys, such as Ti6Al4V [63].

**Figure 2:**
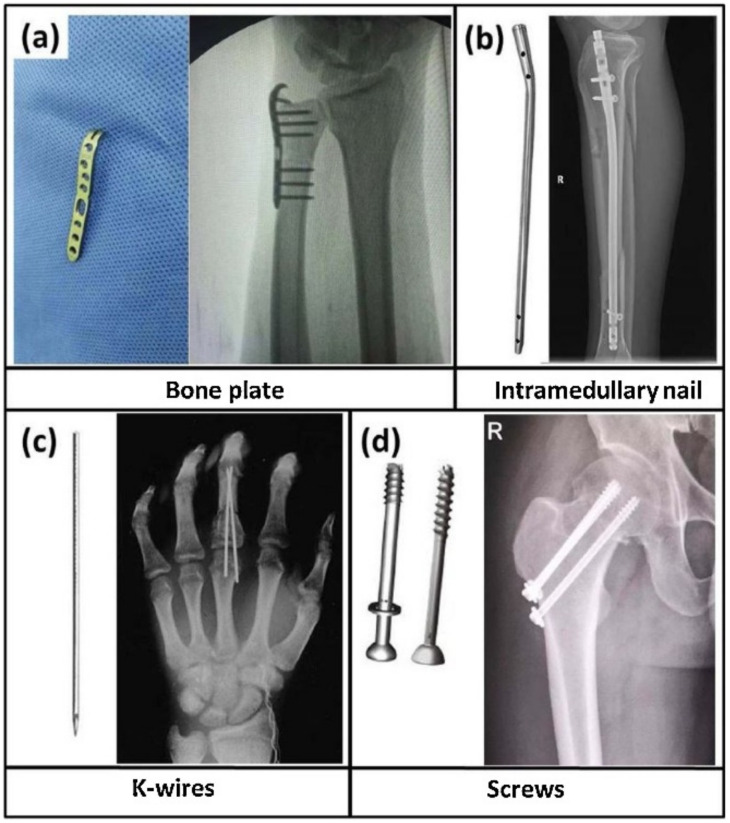
Examples of bone fixation devices: (**a**) a bone plate, (**b**) an intramedullary nail, (**c**) a K-wire, and (**d**) screws. Reprinted from Materials evolution of bone plates for internal fixation of bone fractures: A review, Vol 36, Junlei Li, Ling Qin, Ke Yang, Zhijie Ma, Yongxuan Wang, Liangliang Cheng, Dewei Zhao, Materials evolution of bone plates for internal fixation of bone fractures: A review, Pages No. 190–208, Copyright (2020), with permission from Elsevier [65].

Clips, sutures, and staples are examples of the soft tissue fixation devices used for the surgical fixation of torn connective tissue to the bony insertion site and wound closure [64]. On the other hand, bone fixation devices are required for the external and internal fixation of bone and include plates, screws, wires, pins, and rods (Figure 2) [65].

A universal problem associated with metal implants is their possible corrosion in the human body, which may lead to osteolysis and even to large pseudotumors (large, cystic, or solid masses), thus compromising bone and soft tissue. In the worst case scenario, the corrosion of modular junctions may necessitate the excision of an entire fixed component [31]. Aksakal et al. [66] conducted a study by investigating Ti6Al4V and 316L steel implants obtained from revision operations between 1993 and 2002. They found that 42% of failures occurred due to corrosion.

Coating the metal surface has been identified as a promising approach to overcoming the challenges associated with metal implants and improving their performance in highly corrosive body fluids [13,67]. If properly selected, multifunctional bioactive coatings could even mitigate the corrosion process. Still, the opposite could also happen (i.e., the bioactive coating can increase the corrosion susceptibility of the metal substrate), which is why obtaining information on the corrosion properties of the functionalized metal material is of utmost importance.

### 3D-Printed Biocompatible Metal Materials

**Figure 3:**
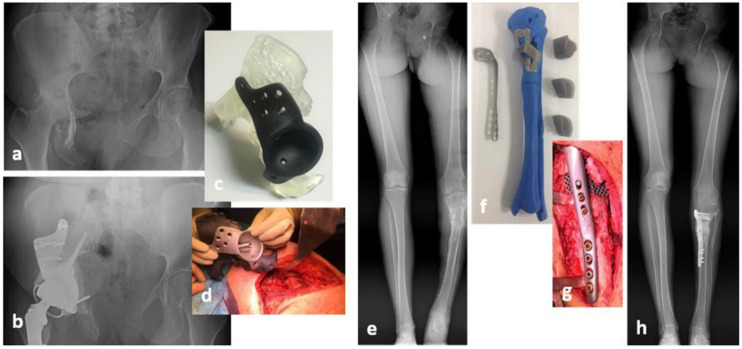
Customized pelvic (**a**–**d**) and tibial (**e**–**h**) implants. Reproduced from Calvo-Haro, J.A., Pascau, J., Mediavilla-Santos, L. et al. Conceptual evolution of 3D printing in orthopedic surgery and traumatology: from “do it yourself” to “point of care manufacturing”. BMC Musculoskelet Disord 22, 360 (2021) [71]. This is an open access article distributed under the terms of the Creative Commons CC BY license published by Springer Nature.

The development of 3D printing made it possible to produce metallic implants with controlled porosity and a modulus closely matching that of the native bone [68,69]. Moreover, when integrated with the computer-aided design (CAD) technique, 3D printing enables the development of custom-made metallic implants that can fit specific tissue defects, fulfilling the need for implant systems that take into account the patient-specific pathology and minimize bone loss due to the implantation requirements (see Figure 3) [70].

Three-dimensional printing enables the fabrication of complex structures, while providing surfaces that more closely resemble the specific anatomy of the bone [72]. Implants fabricated from smooth and solid Ti and CoCr alloys are often associated with limited osseointegration and stress-shielding-induced osteopenia. Three-dimensional printing may not only produce a structure that better resembles the extracellular matrix of healthy tissues, and is thus more amenable to osseointegration, but also notably decreases the elastic modulus of metals and thus minimizes the stress-shielding effect. Three-dimensional printing promises to produce the final complex product in a single process, thus significantly reducing the time and cost of manufacturing. Lastly, the use of customized surgical items has been shown to markedly shorten the surgical time and to enhance the positive medical outcome of the surgery, thus reducing the duration of hospital stays and minimizing the risk of revision surgery [73].

In the fabrication of 3D-printed metal implants in orthopedics, electron beam melting (EBM) and selective laser melting (SLM) are most often used. They both represent a computer-controlled additive manufacturing fabrication process that enables the production of materials in a layer-by-layer manner. These techniques use an energy source to melt and fuse regions of powder particles according to the computer-aided design (hence the name powder bed fusion technologies) [74]. While the energy source in the SLM is a laser beam with an adjustable wavelength, an electron beam is used in the EBM technology. For this reason, EBM is only applicable for the fabrication of conductive metals, whereas SLM is suitable for the processing of ceramics, polymers, and metals [75].

Both above-mentioned powder bed fusion technologies were successfully employed for the fabrication of patient-specific 3D-printed metal implants. Some examples of their clinical applications are presented in Table 4.

Table 4 shows that custom-made orthopedic and trauma devices are mostly fabricated from Ti6Al4V, which is not surprising since Ti alloys have been used in biomedical devices for a long time and represent a starting material for the production of a vast majority of orthopedic implants. Moreover, 3D-printed Ti alloys have outstanding mechanical properties and the ability to be formed into complex shapes [85]. Table 4 also shows that both powder bed fusion techniques (EBM and SLM) are equally represented. Majumdar et al. [63] published an in-depth review of the two processes and presented the differences regarding the characteristics of the EMB and SLM products.

The fabrication of custom-made implants is generally reserved for patients with more complex reconstructive needs, which usually occur due to bone loss. Since the elderly population is at the highest risk of developing pre-existing conditions that require the use of customized surgical implants (e.g., osteoporosis and osteoarthritis), the need for 3D-printing technology in orthopedics is expected to increase significantly. However, the cost-effectiveness of 3D printing remains a concern, particularly when considering its use in a publicly funded healthcare system. Moreover, randomized controlled trials and long-term clinical data are needed to further assess the potential benefits of 3D printing in orthopedics [86].

## 4. Coating Material Selection

Every bare metal implant possesses limited bioactivity. In order to further initiate interaction with the biological tissue and enable better bonding between the bone and the implant, medications and therapeutic agents can be incorporated directly into the implant surface or applied via (biodegradable) polymeric matrixes [87,88]. If properly selected, such polymers can enhance the biocompatibility of the metal implant, mitigate corrosion, and enable the controlled and tunable release of active ingredients [18,89,90,91,92,93,94,95,96,97,98,99].

The initial step in the development of novel materials is the selection of components. In terms of multifunctional orthopedic implants, the selection of the active ingredients governs the selection of the material needed for their delivery. Furthermore, the success of medical implants greatly depends on the nature of the tissue–implant interface, so a tremendous amount of research has been dedicated to improving and controlling this interface by modifying the surface chemistry [100].

Biodegradable polymers are particularly known for their use as a controllable means of delivering different classes of active ingredients in a sustained fashion, thus avoiding the toxicity associated with high and fluctuating concentrations in systemic therapy [101]. Biodegradable polymeric materials have been experimentally used in the field of orthopedics, mainly as components for internal bone fracture fixation [102].

Section 4.1 briefly presents properties of three different groups (i.e., naturally occurring polymers, synthetic polymers, and inorganic materials) of possible components for the preparation of coatings that have the potential to be used for the delivery of the active ingredients described in Section 4.2.1 (antibiotics), Section 4.2.2 (pain medications), and Section 4.2.3 (anticoagulants).

### 4.1. Carrier Materials

#### 4.1.1. Naturally Occurring Polymers

Dextran is a hydrophilic polysaccharide composed of α-1,6-linked d-glucopyranose [103], which has a high water-binding capacity. Due to its solubility in water and in organic solvents, dextran is suitable for being blended with bioactive agents or hydrophobic polymers. It is used in clinical practice as an antithrombotic agent (it reduces blood viscosity) and as a plasma expander [104]. The disadvantages of custom-made dextran are its high cost and nonavailability [105].

Chitin is a natural biopolymer composed of N-acetyl-D glucosamine connected via (1→4) glycosidic bonds. Carboxymethyl chitin (CMCh) is one of the most attractive derivatives of chitin for biomedical applications [106] and mostly refers to 6-O-carboxymethyl chitin. CMCh is pH-sensitive, has moisture-retention abilities, and has an emulsion stabilizing capacity [107]. Homogenous modification of natural chitin to produce CMCh is challenging due to the crystalline structure of chitin and the high degree of acetylation [108].

Chitosan (CHI), a deacetylated form of chitin, is a linear polymer composed of randomly distributed units of N-acetyl-D-glucosamine and D-glucosamine connected via (1→4) bonds. Some studies suggest that it possesses osteoconductive properties [109]. There also exist indications of its pro-thrombotic effects [109]. It is poorly soluble at neutral pH (its solubility in aqueous solutions is pH dependent) and quickly degrades in vivo due to the natural presence of lysozyme, which cleaves (1→4) glycosidic bonds.

Alginate (ALG) is a polysaccharide extracted from marine algae composed of α-L-guluronate and β-D-mannuronate connected via (1→4) glycosidic bonds [110]. It is edible, water soluble, and gelates into softer structures in a physiological environment, which is why it lacks the mechanical integrity required for orthopedic implant coatings used in load-bearing body parts [111].

Pullulan is an exo-polysaccharide, produced by the yeast-like fungus *Aureobasidium pullulans*, composed of α-(1, 6) maltotriose connected via (1→4) glycosidic bonds [112]. In carboxymethyl pullulan (CMP), carboxylate groups are added to the pullulan main chain. CMP is pH and ionic strength sensitive and shows enhanced vascular permeability [113]. Its applications in the development of bioactive coatings for metal orthopedic materials remain limited due to the high cost. Moreover, CMP is a non-osteogenic polymer and does not promote cell proliferation [112].

Collagens are proteins that consist of three polypeptide chains. They are classified into several sub-families, the most numerous being fibrillar collagens. A member of such a class of collagens is a type I collagen, the most abundant protein in the human body, which is found in tissues that are a part of tendons, skin, cornea, bone, lung, and vessel walls [114]. Collagens are also the primary component of the extracellular matrix in tissues [115], where they interact with other biomolecules, which is often exploited in tissue engineering applications. Collagens are highly abundant, thermally stable, and are used in clinical applications as a bone grafting material (OssiMend^®^). The disadvantage of these proteins is a lack of mechanical strength and structural stability upon exposure to an aqueous environment [116].

As the name suggests, carboxymethyl cellulose (CMC) is a derivative of cellulose, a well-known naturally present material composed of β (1 → 4) linked glucosyl units. CMC is a cellulose ether where the cellulose’s hydroxyl groups are substituted with a carboxymethyl group [117]. CMC is used in foods, cosmetics, and pharmaceutical products as a thickener, emulsifier, and coating [118]. The problem associated with CMC is the heterogeneous nature of commercial products due to the uneven degree of substitution [119].

Cellulose acetate (CA) is an acetate ester of cellulose in which the cellulose’s hydroxyl groups are replaced by acetate groups. CA is generally insoluble in water, hydrolytically stable, and chemically resistant [120]. However, the degree of substitution significantly impacts the properties of CA [121].

Hydroxypropylmethyl cellulose (HPMC) is a nonionic biopolymer, a cellulose ether of randomly substituted O-methyl and O 2-hydroxypropyl groups in their anhydroglucose units. HPMC is hydrophilic and suitable for the formation of films. Its applicability remains limited due to its broad dispersity and variation in the degree of substitution (batch to batch) [122].

Hyaluronic acid (HA) is a component of an extracellular matrix in different tissues and is involved in cellular signaling. HA is used in dermal fillers (for example, Juvéderm^®^) and in viscosupplements for arthritis treatment (for example, Durolane^®^). HA easily degrades in the human body due to the natural presence of the enzyme hyaluronidase. Inhibiting its activity requires the use of a hyaluronidase inhibitor [123].

Fucoidan is a sulfated polysaccharide whose main component is L-fucose. It is extracted from different species of brown algae and brown seaweed. Fucoidan is water soluble and forms highly viscous solutions. Despite being used in foods, dietary supplements, and cosmetics, it has not yet been approved for biomedical applications [124]. The disadvantages related to fucoidan are its high cost, source-dependent physicochemical properties, and bioactivity [125].

#### 4.1.2. Synthetic Polymers

Polylactic acid (PLA) is a poly-lactone, an aliphatic polyester derived from lactic acid (2-hydroxypropionic acid). PLA is hydrophobic, thermoplastic, and used in orthopedics as a component of fixation devices. Its challenging synthesis contributes to its high cost [126]. PLA is non-osteogenic [127].

Polyglycolic acid (PGA) is another poly-lactone, an aliphatic polyester derived from glycolic acid (2-hydroxyethanoic acid). PGA has a high melting point and is soluble in most organic solvents. It has been used in clinical applications as a material for absorbable sutures since 1970 (Dexon^®^). High cost and a rapid hydrolysis rate are the two disadvantages of PGA when considering its suitability for the design of multifunctional bioactive coatings.

Polymethyl methacrylate (PMMA) is a vinyl-based polymer with tunable mechanical properties that has been used in clinical applications since the 1950s. PMMA represents the commonest non-metallic implant material in orthopedics [128]. It is nondegradable, non-osteogenic, and may cause necrosis and inflammation [129,130].

Poly(orthoesters) (PEOs) are prepared through the transesterification of orthoesters with diols or through polyaddition between a diol and a diketene acetal [131]. PEOs are hydrophilic and can be used for controlled acid-induced polymer hydrolysis [132]. Biochronomer™ is a PEO developed by Heron Therapeutics, Inc., that is used in clinical practice. The most significant limitation of PEOs is the challenging fabrication of polymers with repeatable characteristics [133].

In ethylene–vinyl acetate copolymers (EVAs), polar vinyl acetate units are randomly dispersed in the backbone. These non-biodegradable polymers are used in a variety of commercial applications, such as stent coatings, intravitreal implants, contraceptive implants, and intravaginal contraceptive rings [134]. SurModics Bravo™ (a blend of poly-butyl methacrylate and polyethylene vinyl acetate polymers) is a product used as a coating for stainless steel Cypher™ stents (Cordis, Santa Clara, CA, USA) that releases the immunosuppressant rapamycin [135].

Polydopamine is obtained by dopamine self-polymerization [136]. It is distinguished by its strong adhesion to most types of surfaces and chemical versatility, which enable combinations with various biomaterials [137]. An important consideration as regards its applicability in implant coatings is its unclear biological effects.

Polycaprolactone (PCL) is a semicrystalline polyester with a very low in vivo degradation rate and high drug permeability that is often blended or copolymerized with other polymers to promote overall polymer erosion [138]. PCL is an FDA-approved biomaterial for biomedical applications [139]. It has been used for the in vivo delivery of levonorgestrel in a commercial contraceptive product, Capronor^®^, for over three decades [140].

#### 4.1.3. Inorganic Coating Materials

Monodispersed silica spheres (SiO_2_) are synthesized using a sol–gel technique and represent a versatile starting material for surface functionalization with organic reagents in order to obtain drug delivery vehicles. This hydrophilic material is tunable in size, has a high surface area, and enables facile surface modifications [141]. Since silica appears to be required for bone formation [142], SiO_2_ shows potential in bone repair applications if aggregation under physiological conditions [143] and poor mechanical properties can be overcome.

Another group of silica-based materials produced by the sol–gel technique is bioactive glass, a material that has been in clinical use for decades. The most well-known representative, i.e., 45S5 Bioglass^®^, with the following composition: 45 wt% SiO_2_, 24.5 wt% Na_2_O, 24.5 wt%, CaO, and 6 wt% P_2_O_5_, has found application in bone defects [144]. Bioactive glass bonds to the living tissue through the formation of a hydroxy-carbonated apatite layer and has the ability to retain bioactivity after implantation, enabling controlled rates of release of ionic dissolution products [145] and other active ingredients. A limitation of this material is its low fracture toughness, making it unsuitable for load-bearing bone repair [146].

Hydroxyapatite (Ca_10_(PO_4_)_6_(OH)_2_) is a predominant inorganic bone component and the most widely used calcium phosphate bioceramic for coatings on metal prostheses [147]. It is highly osteogenic and has been in clinical use for more than 20 years [148]. The brittleness makes hydroxyapatite unsuitable for load-bearing applications.

Graphene oxide (GO) is a material obtained through the oxidation and exfoliation of graphite where single sheets of sp^2^-hybridized C atoms are arranged within honeycomb lattices [149]. GO is a stiffening and anticrack propagation agent in polymer-based composite materials [150]. GO-based materials are cell-adhesive and show promise as a platform for bioactive functionalization, but their biological effects remain unclear.

An interesting yet not fully exploited type of inorganic coatings is metallic materials. These can be either nitinol-based and composites thereof [151,152] or more unusual coating materials such as hardystonite [153]. Some examples of such coatings were recently reviewed by Hussain et al. [154].

Despite tremendous efforts, only a handful of materials have reached clinical applications, namely collagens, HA, PMMA, PEOs, EVAs, hydroxyapatite, and bioactive glass. The potential disadvantages and limitations of the biopolymers and inorganic coating materials presented in this section that are relevant for the design of multifunctional bioactive coatings on metal materials in orthopedics include challenges associated with fabrication, high costs, and, very importantly, a lack of mechanical integrity. The latter is critical for the applicability of such coatings in bone repair, since the coating must not be removed from the surface during the surgical procedure (e.g., THA, total knee replacement, osteosynthesis procedures, etc.). Since not a single component meets all the necessary requirements, scientific groups are studying combinations of different classes of materials to develop coatings with enhanced structural properties [155,156].

When developing a controlled release formulation, the choice of an active ingredient dictates not only the selection of the biodegradable polymeric carrier material but also the manner in which these active ingredients are to be embedded into the coating for an implantable device. In other words, both the dosage and the nature of the released compounds may be controlled by adjusting the chemistry of the degrading material [157]. Release profiles are highly dependent on the chemical composition of the polymer and can be predicted to some extent by obtaining information on the degradation and swelling properties of the embedded polymer. Natural polymers generally enable efficient release for up to a few days [23,158], which is too short of administering all active ingredients in clinically relevant time frames. For example, it takes several months to complete the bone healing process [159], so coatings with incorporated active ingredients that affect this process should enable therapeutic efficiency for a month(s). The addition of synthetic polymers can lengthen release duration and provide coatings with mechanical properties that can withstand handling during orthopedic surgical procedures.

### 4.2. Active Ingredients

#### 4.2.1. The Selection of an Antimicrobial Agent

Orthopedic and trauma-device-related infections remain a significant burden on healthcare systems around the world, even in environments with the best clinical practices [160]. One of the reasons for the difficult treatment of implant-associated infections (IAI) is the insufficient delivery of drugs to sites of infection through systemic administration, since drug concentrations below a therapeutic dosage cannot reduce the formation of bacterial biofilms on implants [161]. Despite the introduction of peri-operative antibiotic prophylaxis as a key measure in preventing surgical site infections (SSI) following orthopedic surgery, significant numbers of septic complications, especially in high-risk patients and procedures, are still being reported [16]. These septic complications are mostly caused by IAI.

For example, periprosthetic joint infection (e.g., hip and knee arthroplasty) remains one of the most severe complications in orthopedic surgery, with infection rates ranging from 0.7% to 4.2% [160,162,163]. What is concerning is that 10% of these treatments will require revision surgery [30]. Similarly, the incidence of infection after surgical procedures that involve internal fixations of bone fractures with implantable devices (such as wires, nails, plates, and screws) ranges from up to 2% for closed fractures to over 30% for open fractures [164]. Rates of postoperative infection after spine surgery vary between 1.1% and 4.4%, depending on the spinal pathology and age of the patient [165], while deep prosthetic joint infections occur in 14% of cancer patients [166].

Competition for surface colonization between the host cells and bacteria occurs every time a biomaterial is implanted. Bacterial adhesion to an implant can cause biofilm formation, making the bacteria extremely resistant to the host’s defense mechanisms [167]. Studies have proven the formation of bacterial biofilms (the rate of their formation is species dependent) only a few hours after the adhesion of the first bacteria on a substrate. Biofilm formation effectively protects microorganisms from the immune system and systemic antibiotics and is therefore the most critical pathogenic event in the development of IAI [16,168]. Hence, the destiny of an implant is decided at the time of surgery when an environment favorable to the host and hostile to microorganisms should be created [15].

According to the literature, Gram-positive cocci cause up to 87% of implant-related infections, whilst up to 17% of such infections were reported to be caused by Gram-negative bacilli (see Table 5).

Table 5 shows that a wide array of bacteria is responsible for orthopedic implant-related infections. Therefore, broad-spectrum antibiotics are used in clinical practice, such as cephalosporins, co-amoxiclav (a mixture of amoxicillin and clavulanic acid), clindamycin (CLIN), quinolones, teicoplanin, vancomycin (VAN), and gentamycin (GEN) [175,176,177]. The selection of prophylactic antibiotics depends on the type of surgery, the characteristics of the environment (some geographic areas have a high prevalence of *S. aureus*, whereas *S. epidermidis* is more common in others [178]), and of course, the type of patient. For example, chronic patients in long-term institutional care are much more likely to be colonized with MRSA, which is why such patients receive treatment with an antibiotic sensitive to this type of microorganism [179]. Therefore, the end goal in the development of antibacterial biomaterials should be to enable personalized prophylaxis tailored to the specific risk factors of each patient by enabling local therapy for a wide range of antibiotics.

A solution for the local delivery of antibiotics in high concentrations has been available in clinical orthopedic practice for decades. Antibiotic-loaded PMMA (commonly known as bone cement) is a nondegradable biomaterial that can be used in primary and revision arthroplasty to treat prosthetic joint infections and treat chronic osteomyelitis [180]. The mechanical strength of PMMA is satisfactory, but the efficiency of the local drug release remains questionable [181]. Another disadvantage of PMMA is that not all types of antibiotics are suitable for incorporation, and their delivery is not tunable. Lastly, there exist concerns about possible implant failure due to PMMA debris-induced biological reactions [182].

**Figure 4:**
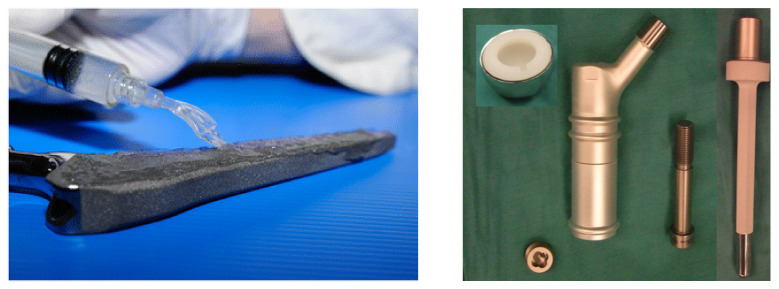
**Left**: DAC^®^ hydrogel coating spread onto a cementless hip prosthesis. Reproduced with permission from *Journal of Orthopaedic Surgery and Research* under the terms of the Creative Commons Attribution 4.0 International license (CC BY 4.0). Carlo Luca Romanò, Sara Scarponi, Enrico Gallazzi, Delia Romanò, Lorenzo Drago, Antibacterial coating of implants in orthopaedics and trauma: a classification proposal in an evolving panorama, Vol 10, Copyright (2015) published by Springer Nature [16]. **Right**: A new silver-coated prosthesis. Reproduced with permission from *BioMed Research International* under the terms of the Creative Commons Attribution 4.0 International license (CC BY 4.0). F. Donati, G. Di Giacomo, S. D’Adamio, A. Ziranu, S. Careri, MA. Rosa, G. Maccauro, Silver-coated hip megaprosthesis in oncological limb savage surgery, Vol. 2016, Copyright (2016) published by Hindawi [185].

Among coatings, only three antibacterial technologies are currently present on the market: silver coatings, GEN poly(D, L-lactide) (PLLA) coating, and a fast-resorbable hydrogel coating composed of covalently linked hyaluronan and PLLA (Defensive Antibacterial Coating; DAC^®^; Novagenit Srl, Mezzolombardo, Italy) [15]. DAC^®^ (see Figure 4 Left) has been available for clinical use since 2013 [183]. While studies demonstrating its efficiency in preventing different types of implant-induced infections exist, studies with a greater number of patients and longer follow-up are lacking. The limitations of this technique are that it enables the addition of a limited amount of antibiotics and its considerable cost [184]. In addition, the gel-like structure of DAC^®^ does not provide the mechanical strength and structural stability required for applications in bone repair, causing its unintentional removal during the surgical procedure.

GEN PLLA fully resorbable matrix is a coating for tibial nails loaded with GEN, whose biggest limitation is that it is only applicable for a single type of bone, a single type of surgical instrument, and enables the release of a single antibiotic.

Despite the existence of silver-coated prostheses (see Figure 4 Right) produced by several manufacturers, such as Agluna (Accentus Medical Ltd., Didcot, United Kingdom), Mutars (Implantcast GmbH, Buxtehude, Germany), and PorAg (Waldemar Link GmbH & Co. KG, Hamburg, Germany), there is still concern about the toxicity of silver, which is why the routine use of silver-coated implants remains limited. Furthermore, studies on silver implant coatings have been unable to disprove possible detrimental effects on osseointegration or to demonstrate sustained controlled release above minimum inhibitory concentrations in vivo [186].

Since it is impossible to ensure completely sterile conditions during (orthopedic) surgical procedures, preventing the occurrence of implant-related infections is as important as the treatment itself. The local application of antibiotics at optimal concentrations over appropriate stages of surgery and recovery would avoid the disadvantages of the traditional systemic drug treatment of IAI by enhancing treatment efficiency and diminishing potential drug-specific toxicities [101].

Data about the ideal release profiles of antibiotics from surface coatings in the literature are contradictory. Some researchers state that the aim in coating design should be the release of antibiotics at optimal bactericidal levels to prevent potential infection. In contrast, afterwards, the antibiotic release should cease quickly to minimize any unwanted effects of antibiotics on the implant’s tissue integration [181]. On the other hand, some scientists argue that coatings should enable long-term release [187] to prevent biofilm formation and late-stage infections by providing a sustained drug release (above the minimum inhibitory concentration) that continuously eradicates newly grown bacteria [188]. The ideal release profile of antibiotics from surface coatings probably depends on a lot of factors, such as the antibiotic’s mechanism of action, the rate of bacterial colonization, and the presence of other active ingredients, so the answer to this question is not straightforward. What is certain is that the applicability of bioactive coating materials in clinical practice calls for the introduction of guidelines for local delivery and the establishment of the corresponding drug and implantation site-specific pharmacodynamic principles [160].

#### 4.2.2. The Choice of Pain Medications

Current clinical practice includes peri-operative antibiotic prophylaxis and postoperative pain management [189]. Aside from being a source of great discomfort to the patient, untreated pain can slow rehabilitation after orthopedic surgery, thus contributing to a longer hospital stay, unplanned re-admissions, and increasing the cost of care [190].

Orthopedic surgical trauma commonly results in neuropathic pain due to nerve damage in the implant vicinity during surgical procedures. For this reason, a combination of different methods of pain control (also termed multimodal pain management) is preferred [191]. In multimodal pain management, painkillers with different mechanisms or sites of action are combined. This strategy is based on the synergistic effects of the drugs and provides improved analgesia with reduced unwanted side effects [192]. Nowadays, multimodal therapy is standard clinical practice, an indispensable tool in postoperative pain management.

Pain treatment after orthopedic surgery typically involves the intravenous administration of medications and the usage of tablets that are taken orally. These methods are often associated with systemic adverse effects and do not enable the delivery of pain-relieving agents to a specific area in the human body. Implementing controlled tunable delivery of biodegradable carrier materials embedded with pain medication(s) for postoperative pain management could enable local treatment for a pre-programmed amount of time. Using this approach, many of the common unwanted side effects of pain medication (see Table 6) would be limited as the drug would only be exposed to the implantation tissue [158].

Currently, the drugs commonly used for postoperative pain treatment after orthopedic procedures are nonsteroidal anti-inflammatory drugs (NSAIDs) and anti-neuropathic pain drugs (e.g., gabapentin, pregabalin). The latter are used either individually or in combination with NSAIDs. Examples of recommended multimodal analgesic regimens after commonly performed types of orthopedic surgery are presented in Table 6.

**Table 6:** Examples of the drugs used in multimodal pain management in commonly performed orthopedic surgeries [193].

Drug Class	Examples	Contraindications and Cautions in Systemic Delivery	Total Hip Replacement	Total Knee Replacement	Spinal Fusion
**NSAID**	Ketorolac, ibuprofen, meloxicam, diclofenac	Gastrointestinal bleeding and ulceration, cardiovascular events, renal dysfunction	YES	YES	NO
**Anti-neuropathic**	Gabapentin	Dizziness, sedation; reduced dose with renal dysfunction	Gabapentin OR pregabalin	Gabapentin OR pregabalin	Gabapentin OR pregabalin
	pregabalin				
**Analgesic and antipyretic**	Acetaminophen, paracetamol	Hepatotoxicity	AND/OR	AND/OR	YES
**Local anesthetic**	Lignocaine, bupivacaine, ropivacaine, prilocaine [194]	Local anesthetic systemic toxicity (LAST), methemoglobinemia [195]	YES	YES	NO

Several functionalized model implants containing pain medications are being investigated for their applications in the field of pain management in orthopedic procedures, but none has gained regulatory approval thus far. There exist, however, a few products in the form of (injectable) solutions that provide the local release of analgesics. The newest example of such a product on the market is the extended-release solution ZYNRELEF™ (bupivacaine and meloxicam), which was approved by the FDA as a dual-acting local anesthetic for pain management following total knee arthroplasty and some other types of surgery. As of 1 January 2021, ZYNRELEF™ has also been approved in the European Union and the United Kingdom [196]. Bupivacaine is an anesthetic, while the NSAID meloxicam reduces the local inflammatory response to tissue injury. A 72 h release of both pharmaceuticals is provided by a polymer technology based on a group of poly(orthoesters) developed by Heron Therapeutics, Inc., San Diego, California, United States (Biochronomer™) [197]. Since the polymer formulation is viscous and hydrophilic, the formulation can be applied to the affected tissue in the surgical incision without a needle and remains where it is placed, thus releasing both drugs simultaneously [197].

Although sustained-release formulations of local pain-relieving agents represent a promising strategy for pain management after orthopedic surgery, more research is required to maximize their potential. Above all, it is necessary to develop a coating system that will enable better control over the release of drugs and thus effective pain therapy tailored to each patient’s individual needs.

#### 4.2.3. A Presentation of Anticoagulation Agents

Deep vein thromboses (DVT) and pulmonary embolisms (PE) are subsets of venous thromboembolism (VTE) and are responsible for significant morbidity and mortality in the population worldwide. DVTs develop when a blood clot forms in a deep vein, usually in the thigh, pelvis, or lower leg. In the United States alone, emergency clinicians diagnose DVTs in approximately one out of every 500 adult emergency department patients [198]. PEs occur when a blood clot dislodges and travels through the bloodstream to the lungs. Among acute cardiovascular diseases, PEs have been identified as the third greatest contributor to mortality, with a nearly 30% mortality rate [199].

VTEs are a complication of major orthopedic surgeries (e.g., total hip replacement (THR), total knee replacement (TKR), and hip fracture (HF)), which can have devastating consequences, as illustrated by the 29.4% mortality rate in HFs within 30 days of a VTE event. It is estimated that without prophylaxis, about 5% of patients undergoing a THR, TKR, or HF could develop a symptomatic VTE [200]. Of these, 0.2% to 1.1% will develop a PE within 35 days of surgery, as the first postoperative week carries the highest risk of the occurrence of a PE [199]. The treatment of orthopedic-related VTEs represents a substantial economic and financial burden, assessed as amounting to USD 5000 after 3 months, USD 10,000 after 6 months, and USD 33,000 after 1 year [201].

Elderly patients usually have various comorbidities, which puts them at a much greater risk of developing a VTE during and after hospitalization. Moreover, following hospitalization, many elderly patients continue to have limited mobility. Those discharged to nursing home care are at up to a 30-fold higher risk of developing a VTE than the general population [202].

Venous thrombi are intravascular deposits of fibrin, combined with erythrocytes, platelets, and leukocytes [203]. They are mainly formed in regions of slow or disturbed blood flow due to an increase in the concentration of locally activated coagulation factors. The decreased velocity and mechanical alterations of blood flow influence the interaction between the endothelial lining of the vessels and the components of blood to create a hypercoagulable state. This condition is associated with every type of major surgery (including THR, TKR, and HF) and persists into the postoperative period [203].

**Figure 5:**
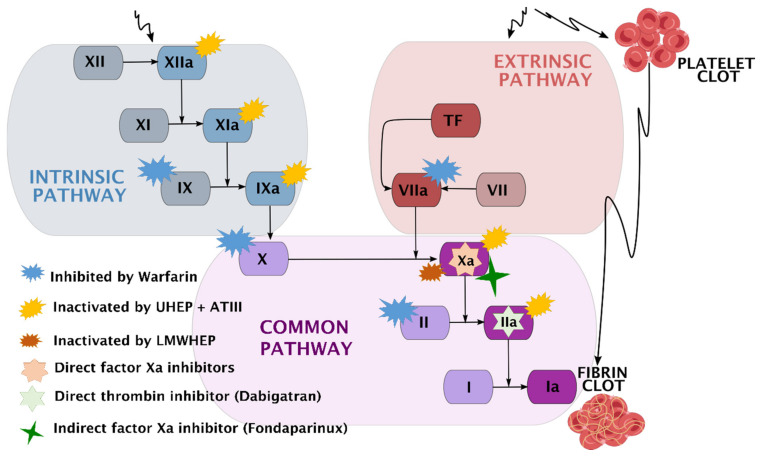
An overall presentation of the coagulation pathway and the sites of action of anticoagulants used in clinical practice. The coagulation factors are represented by Roman numerals, and the letter “a” indicates their activated form. Adopted after Dwivedi and Pomin [207], Gando et al. [208], and Jay and Lui [209].

Thrombosis leads to the formation of an occlusive blood clot and is essentially an exaggerated hemostatic response [204]. The term hemostasis describes an enzyme cascade of activation reactions (called a coagulation pathway) that leads to the blockage of bleeding. In the primary stage, the damaged endothelial cells are protected by an aggregation of platelets (forming a plug), whereas the end goal of the secondary stage of hemostasis is to stabilize this platelet plug with a fibrin mesh. Secondary hemostasis can be divided into two main coagulation pathways: an intrinsic pathway and an extrinsic pathway. They originate separately but converge at a specific point to form a common pathway, activating fibrinogen into fibrin. Fibrin molecules can be viewed as building blocks forming fibrin strands, which stabilize the platelet plug by binding them together [205]. An overall presentation of the coagulation pathway is outlined in Figure 5.

Due to the reasons given above, systemic thromboprophylaxis is nowadays a standard of care in major orthopedic surgeries. The selection of an anticoagulant depends on a thorough risk assessment of the patient, where the need to minimize VTE needs to outweigh the undesirable (and occasionally fatal) consequences [206]. Table 7 presents a list of the anticoagulants used in clinical practice, along with their pharmacodynamic and pharmacokinetic properties, while Figure 5 illustrates their sites of action.

Heparins (HEPs) have been used in clinical practice as anticoagulants for decades. HEPs are classified into unfractionated heparins (UFHs) and low molecular weight (MW) HEPs. The first is a glycosaminoglycan consisting of long linear chains (MW_avg_ approx. 12 kDa) of variably sulfated disaccharide units of 1,4-linked uronic acid and glucosamine residues [215], whereas the latter represents a depolymerized version of UFH (MW_avg_ < 8 kDa). The non-eluting HEP coatings compatible with metal substrates are already commercially present (CORLINE^®^ manufactured by Corline Biomedical AB, Uppsala, Sweden, Hepamed™ manufactured by Medtronic, Minneapolis, Minnesota, United States, and PHOTOLINK^®^ manufactured by Surmodics, Inc., Eden Prairie, Minnesota, United States). However, covalent immobilization of HEP does not guarantee its anticoagulant activity [216], so systems that enable a controlled release of HEP and consequently its therapeutic efficiency remain an extensively researched topic.

An increased risk of bleeding is the major complication of anticoagulation prophylaxis and represents a serious safety concern. Moreover, anticoagulants that are primarily eliminated by the kidneys (e.g., LMWHs, direct factor Xa inhibitors) can accumulate in (mostly elderly) patients with impaired renal functioning. Consequently, clinicians generally evaluate kidney functioning in all patients commencing anticoagulant therapy [211]. Furthermore, the already-mentioned polypharmacy present in many elderly patients additionally contributes to the incidence of complications through drug interactions either via drug metabolism or via excretion [217].

As stated above, aging is one of the strongest risk factors for VTE. Nearly 60% of VTE events occur in the population over 70 years old. From our mid-twenties to our mid-eighties, the overall incidence of a VTE event increases by 80-fold [218]. Moreover, advancing age is associated with a significant increase in major bleeding episodes [219].

Localized, controlled, and continuous release of anticoagulants would prevent clotting on synthetic surfaces without systemic effects. This could offer a superior solution to thromboprophylaxis while fulfilling the need for improved orthopedic implants [220]. However, to obtain a clear answer for the benefit of patients, additional evaluation of the potential advantages of local anticoagulant delivery over systemic therapy is needed in the future.

## 5. The Latest Strategies in the Development of Multifunctional Bioactive Coatings

This section is intended to provide a brief overview of the experimental results and corresponding references in order to aid researchers in identifying some of the latest strategies in the development of multifunctional bioactive coatings that address the bioactivities presented in Section 4 by employing clinically relevant pharmaceuticals (see Table 8).

The data presented in Table 8 show that the majority of recently developed coatings were applied on Ti-based and stainless steel substrates, while nearly half of the research presents a coating with at least one incorporated antibiotic. Several research groups have combined antimicrobial and osteointegrative activity into a single coating system in order to develop solutions that might aid in preventing aseptic implant loosening, a common cause of orthopedic revisions worldwide. A promising approach to enhancing implant fixation is the application of osteoinductive growth factors that promote new bone formation [238,239]. Bone morphogenetic protein 2 (BMP-2) is probably the most widely present osteoinductive growth factor in the development of bioactive coatings since it has obtained clinical approval [240]. However, when introduced to the market, reports of complications and unwanted side effects emerged due to local overdoses and unsustained release, shifting the focus of the research community to the optimization of therapeutic efficacy and the safety of BMP-2 by developing systems that enable the localized delivery of lower doses and prolong the retention time at the site of action [241].

It has been published that simvastatin (SIM), a well-known medication used to help lower cholesterol, could also be used to promote osteogenesis [242]. Nonetheless, further investigation is necessary to fully confirm the osteogenic capability of SIM.

Contradictory to the general glucocorticoid mechanism of action, some research findings suggest that dexamethasone (DEX), usually used for the treatment of inflammatory and autoimmune diseases, might promote osteogenesis at physiological concentrations [243]. For this reason, it was studied as an active ingredient to prepare bioactive coatings intended to improve the osseointegration at the interface between the implant and the bone.

CHI and HEP were the most frequently selected components for the preparation of carrier matrixes. These two groups of polymers have been extensively studied for drug delivery applications due to their biocompatibility, biodegradability, cost-effectiveness, and chemical versatility [244,245]. Another frequently employed group of polymers are polydopamines, known for their facile and cost-effective fabrication, excellent biocompatibility, and multi-drug carrier capacity [246,247].

A potential concern that needs to be addressed in the development of bioactive coatings that enable the release of multiple pharmaceuticals is the compatibility issues and potential drug–drug interactions that might occur during the local delivery of all of the active components. Furthermore, the clinically relevant dosing in systemic therapy is not necessarily relevant for local delivery and most probably requires different concentrations and therapeutic time frames. This is probably the reason why only four articles in Table 8 report on the incorporation of more than one pharmaceutical.

While all the studies presented are of high quality and value to the scientific community, there exist certain universal limitations as regards the potential applicability of the newly developed materials. Firstly, the bioactive coating must be stable under the physiological stress associated with locomotion and must not detach from the implant surface [248]. Secondly, if the coating technology is not suitable for implementation near or in the operating room, the coated device should be able to withstand conventional sterilization techniques without damaging the incorporated drug [8]. Thirdly, the coating of the metal surface might increase its susceptibility to corrosion, thus obtaining information on the corrosion properties of the functionalized metal material should be thoroughly evaluated.

## 6. Concluding Remarks and Outlook

The aging of the population will be accompanied by an increased number of orthopedic surgeries and a rise in the incidence of complications, while the implanted metal materials will be exposed to a higher risk of failure due to the longer indwelling times and the medical complexity of the elderly patients. For this reason, enhancing the performance of metal orthopedic implant materials to increase their lifespan and to prevent the occurrence of revision surgeries is of utmost importance and presents a rationale for the systematic development of materials that enable personalized treatment. Since metals cannot be replaced by ceramics or polymers due to their mechanical superiority, they will continue to dominate the orthopedic implant market for the next few decades. Therefore, future improvements need to combine mechanically superior metals with enhanced biocompatibility and biofunctionality in order to develop implants with the best possible medical performance. The necessary bioactivation of bare metal-based implants can be achieved by multifunctional bioactive coatings that aim to combine multiple medications within the same implanted material for synergistic results while keeping other body parts unaffected, thus avoiding potentially serious unwanted systemic side effects.

Successful innovation in biomaterial design requires a detailed understanding of the properties and mechanisms of biological matter. Despite continuous advances in understanding the natural functioning of biological materials and systems in the development of multifunctional orthopedic metal materials, a cohesive and systematic approach is still missing. Understanding the interaction of materials with “living” tissues remains one of the critical obstacles in biomaterial development. An in vitro testing platform that would enable an accurate evaluation of a material’s functioning in vivo would accelerate progress towards applying novel multifunctional bioactive materials. To achieve this goal in the not-too-distant future, the exact dosing, its tunability, predictable pharmacokinetics, the adoption of stability tests, and the development of systematic guidelines are essential.

The translation of such research into clinical practice is extremely challenging, since the surface treatment must be not only efficient but also cost-effective. The scalability of the components used for the surface treatment is one of the factors that is often overlooked in the development process. Coatings with few clinical benefits that significantly raise the price of an implant will never be produced on an industrial scale. Moreover, a multifunctional bioactive coating system that requires a fundamental change in the manufacturing process is likely to notably shorten the implant’s shelf life, putting into question its commercial viability, so the coating technology should possibly be applied near or in the operating room.

We believe that the successful development of such materials is being hindered by the challenging preparation of formulations with several different pharmaceuticals, the lack of extensive knowledge of the chemical interactions between the components, and the fact that even minor variations in the content and composition of bioactive coatings may have a profound impact on their performance.

The long-term aim in the development of multifunctional bioactive orthopedic implants is to develop materials exactly customized to an individual’s anatomy that enable efficient patient-specific pharmacotherapy. If used in clinical settings in the future, such materials would greatly increase the success rate of orthopedic surgical implantations and prolong material lifetime, leading to a significant decrease in morbidity and mortality.

## Figures and Tables

**Table 1:** An overview of the European statistics on the frequency of hip replacement and the frequency of total knee replacement in 2018, as provided by Eurostat [28].

Country	Hip Replacements per 100,000 Inhabitants	Total Number of Hip Replacements	Knee Replacements per 100,000 Inhabitants	Total Number of Knee Replacements
Belgium	274.6	31,303.3	207.3	23,626.5
Bulgaria	117.7	8241.8	31.5	2207.8
Czechia	199.2	21,115.2	144.7	15,337.1
Denmark	241.4	13,952.3	181.2	10,471.0
Germany	310.6	257,129.2	222.8	184,431.3
Estonia	170.2	2246.6	108.3	1429.8
Ireland	123.3	5953.0	47.5	2295.2
Spain	121.5	56,691.9	132.2	61,675.2
France	248.6	166,315.3	181.8	121,612.7
Croatia	171.0	7012.6	72.8	2984.8
Italy	184.9	111,815.4	128.9	77,952.7
Cyprus	55.5	660.0	54.4	646.3
Latvia	180.4	3488.4	103.9	2010.0
Lithuania	200.6	5616.8	124.4	3484.0
Luxembourg	181.6	1089.5	182.1	1092.5
Hungary	138.8	13,466.5	88.6	8598.1
**Malta ^1^**	88.9	391.1	167.3	736.2
Netherlands	222.3	37,975.7	171.4	29,282.0
Austria	298.5	26,332.8	229.9	20,284.4
Poland	161.8	61,444.0	66.8	25,385.8
**Portugal ^2^**	90.6	9397.3	62.2	6448.1
Romania	71.4	13,936.6	24.7	4816.1
Slovenia	187.7	3753.4	132.8	2655.8
Slovakia	129.0	7094.5	105.9	5822.3
Finland	274.5	15,097.5	233.4	12,838.7
Sweden	242.0	24,487.4	130.6	13,213.7
United Kingdom	187.1	123,964.7	148.4	98,371.2
Liechtenstein	26.2	9.9	7.8	3.0
Norway	259.6	13,863.2	130.7	6979.9
Switzerland	307.3	26,118.8	250.2	21,265.3
Total	5466.6	1,069,964.7	3874.6	767,957.5

^1^ Data refers to year 2017; ^2^ Data refers to year 2015.

**Table 2:** Estimated hospital costs for revision surgery after hip or knee prosthetic joint infection in Europe and the United States, adopted after Romanò et al. [15]. The number of hip replacements and total knee replacements for European countries were obtained from Table 1. The approximate costs were then calculated by multiplying the data given below.

Condition	Country	Estimated Cost per Patient	Ref.	Total Number of Hip Replacements	Approximate Costs of Revision in 1% of the Population (Millions)
**Hip prosthetic joint infection**	France	EUR (23,757 ± 8235)		166,315	EUR (40.0 ± 13.7)
	Italy	EUR (60,394 ± 15,886)	[38]	111,815	EUR (67.0 ± 1.88)
	Germany	EUR 20,166	[37]	257,129	EUR 51.9
	United Kingdom	GBP (21,937 ± 10,965)	[40]	123,965	GBP (27.2 ± 13.6)
	United States	USD 31,753	[34]	438,000 [39]	USD 139.0
		USD 30,300	[36]		USD 132.7
		USD 31,312	[32]		USD 137.1
**Condition**	Country	Estimated cost per patient	Ref.	Total number of knee replacements	Approximate cost of revision in 1% of the population (millions)
**Knee prosthetic joint infection**	Germany	EUR 25,194	[33]	184,431	EUR 46.5
		EUR 19,010	[37]		EUR 35.1
	United States	USD 25,692	[34]	686,000 [40]	USD 176.2
		USD 25,300	[36]		USD 173.6

**Table 3:** An overview of the metallic materials used in orthopedic devices cleared or approved by the FDA (adapted from the U.S. Food and Drug Administration, “Biological Responses to Metal Implants” [62]), with some commercial examples.

Device Type	Material	Device Type	Material
**Bone fixation devices**	Ti	**Soft tissue** **fixation devices**	Ti
	Ti6Al4V		Ti6Al4V
	Stainless steel		Stainless steel
	NiTi		Ta
**Prostheses**	Ti		NiCo
	CoCr alloys		NiTi
	Stainless steel		CoCr alloys
**Material**		**Commercial examples**	
Ti		Ti	
		STIKTITE	
Ti6Al4V		Ti6Al4V	
		Regenerex^®^	
		4WEB Medical Truss Implant Technology^®^	
Stainless steel		CarTech^®^ BioDur^®^ 108 Alloy	
Ta		Trabecular Metal™	
CoCrMo		CoCrMo	
		Freedom CoCr^®^	

**Table 4:** Some examples of the clinical application of patient-specific 3D-printed metal materials in orthopedics.

Material	Processing	Application	Patient(s)	Reference
**Ti6Al4V**	EBM	Vertebral body replacement	A 12-year-old boy	[76]
**Ti6Al4V**	EBM	Upper cervical spine reconstruction	2 males and 7 females, 12 to 59 years	[77]
**Ti**	Not specified	Pelvic tumor resection	A 65-year-old male	[78]
**Ti6Al4V**	Not specified	Severe foot and ankle trauma	A 46-year-old female	[79]
**Ti6Al4V**	SLM	Orbital wall injury	A 67-year-old male	[80]
**Ti6Al4V**	SLM	Large cranial defect	A 22-year-old male	[81]
**Ti**	Not specified	Complex midfacial defects	A 50-year-old male	[82]
**Ti6Al4V**	EBM	Wrist arthroplasty	A 34-year-old male, a 39-year-old male	[83]
**Ti6Al4V**	SLM	Upper maxilla waferless repositioning	10 patients	[84]

**Table 5:** A list of microorganisms known to be causing implant-associated infections (IAI) in orthopedic practice and examples of antibiotics that efficiently control against the corresponding class of microorganism.

Commonly Isolated Class of Microorganisms ^1^	The Commonest Species	Sensitivity	Approximate Percentage of Infections Caused	Ref.
**Gram-positive cocci**	Staphylococcus aureus *, *Staphylococcus epidermidis*, *Streptococcus* species, *Enterococcus* species	β-lactams (flucloxacillin, cephalosporins, carbapenems), glycopeptide antibiotics (vancomycin, teicoplanin), lincosamide clindamycin, fluroquinolones, aminoglycoside rifampicin	65	[30]
			54–83	[169]
			44–87	[160]
			64–82	[164]
			70	[170]
**Gram-negative bacilli**	*Enterobacteriaceae*, *Pseudomonas aeruginosa*	Usually, a combination of a β-lactam (e.g., carbapenem) and an aminoglycoside or fluoroquinolone [171]	6	[30]
			10–17	[169]
			6–17	[160]
			8	[164,170]
**Anaerobes**	*Propionibacterium* species, *Peptostreptococcus* species, *Finegoldia magna*	Metronidazole, carbapenems, chloramphenicol, combinations of penicillin and a beta-lactamase inhibitor, tigecycline and clindamycin [172]	4	[30]
			2–4	[169]
			4–5	[160]
**Multispecies bacterial infections**	Various combinations of bacteria (for example: *S. aureus* and *Streptococcus agalactiae* [173] or *Propionibacterium acnes* [174])	Species-dependant	20	[30]
			10–20	[169,160]
			10–12	[164]

^1^ Classification adopted after Del Pozo et al. [30]; * methicillin-resistant *Staphylococcus aureus* (MRSA) is resistant to β-lactams but sensitive to glycopeptide antibiotics, GEN, and rifampicin [175].

**Table 7:** A list of anticoagulants used in clinical settings.

Anticoagulant	Mode of Action	Disadvantages	Administration
**Warfarin**	Inhibits several coagulation factors (II, VII, IX, and X)	Constant blood monitoring is required; interactions with multiple foods and drugs	Oral
**Unfractionated HEP** **(UHEP)**	Binds to antithrombin III (ATIII), inactivating coagulation enzymes XIIa, XIa, IXa, Xa, and thrombin (factor IIa)	Blood monitoring is required; extended use might cause delayed healing, thrombocytopenia, and osteoporosis; [210] variable pharmacokinetic properties [211]	Intravenous infusion (IV) or subcutaneous (SC) injection
**Low molecular weight heparin (LMWHEP)**	Indirect factor Xa inhibitor	Similar to UH but to a lesser extent [210]; dosing depends on creatinine clearance (eliminated by the kidneys)	SC injection
**Dabigatran**	Direct thrombin (factor IIa) inhibitor	May increase the risk of hemorrhagic stroke [212] and gastrointestinal bleeding [213]	Oral
**Rivaroxaban**	Direct factor Xa inhibitors	Dosing depends on creatinine clearance (eliminated by the kidneys)	
**Apixaban**			
**Edoxaban**			
**Betrixaban**			
**Fondaparinux**	Indirect factor Xa inhibitor	Increased risk of major bleeding [214]	SC injection

**Table 8:** A summary of the recently developed bioactive coatings on metal substrates that address some of the bioactivities outlined in Section 4.

Metal Substrate	Carrier Matrix	Active Ingredient	Results	Testing Model	Ref.
**Ti**	HEP/dopamine	HEP	A possible alternative to long-term application in physiological fluid if the anti-erosion capability of the outermost HEP layer could be improved	In Vitro	[221]
**Ti**	Hydroxyapatite-HEP	HEP	Homogeneous incorporation of HEP in the composite films and enhanced bioactivity	In Vitro	[222]
**Ti6Al4V**	A partially sulphated HA functionalized with a dopamine moiety	GEN and VAN	Demonstrated prevention of biofilm formation on the surface of the Ti alloy samples	In Vitro	[223]
**Ti**	Polydopamine coating followed by the deposition of the GO coating loaded with HEP	HEP	Improved blood compatibility of Ti, the promotion of endothelial cell adhesion and proliferation	In Vitro	[224]
**316L stainless steel**	Polyglycidyl methacrylate grafted with HEP/NONOate nanoparticles	HEP	Improved anticoagulation, anti-restenosis, and enhanced endothelial regeneration	In Vivo	[225]
**Ti**	HEP-grafted surface	Alendronate	Dual bioactivity: enhanced osteoblast differentiation and inhibited osteoclast differentiation	In Vitro	[226]
**Ti**	GO	HEP	The coating improved hemocompatibility and cytocompatibility with endothelial cells	In Vitro	[224]
**Ti**	Hydroxyapatite	HEP and BMP-2	Sustained release of BMP-2 from the coating, increased bone formation, and osseointegration	In Vitro and in vivo	[227]
**Ti**	GO	Aspirin	Enhanced osteoblast proliferation and osteogenic differentiation, sustained release of aspirin for 3 days	In Vitro	[149]
**316L** **stainless steel**	Gelatin nanospheres/CHI	DEX	Inhibited inflammation and stimulated osteogenesis, sustained release of DEX for up to 28 days	In Vitro	[228]
**Ti6Al7Nb**	Polylactic-co-glycolic acid, dipalmitoyl phosphatidyl choline, and distearoyl phosphatidyl choline	Doxycycline	Protection against doxycycline-resistant MRSA, release of doxycycline for up to 28 days	In Vitro and in vivo	[20]
**CoCrMo**	Silk fibroin	GEN	Enhanced initial osteoblastic response on coated substrates, antibacterial effect within 1 week	In Vitro	[229]
**Ti**	Ca-P	Simvastatin (SIM) and metronidazole (MNZ)	Controlled release of both SIM and MNZ, increased osteogenic cell differentiation, and the inhibition of bacterial growth	In Vitro	[230]
**AISI 316L** **stainless steel**	CHI/bioactive glass	GEN	Sustained drug delivery over a period of 8 weeks, inhibited bacterial growth for the first 2 days, and support of cellular proliferation for up to 10 days	In Vitro	[231]
**Ti6Al4V and** **316L stainless steel**	Phosphatidylcholine coatings loaded with either one or both of the antibiotics	Amikacin and VAN	The eluted antibiotics showed prevention of biofilm formation	In Vitro and in vivo	[232]
**Ti**	Polydopamine	Cefotaxime sodium (CS)	The CS-grafted Ti substrate was biocompatible, haemocompatible, and could effectively prevent adhesion and the proliferation of *E. coli* and *S. mutans*	In Vitro	[233]
**Ti6Al4V**	Collagen/hydroxyapatite layers	VAN	The coating enhanced osteointegration; local VAN release 7 days following implantation	In Vitro and in vivo	[19]
**Ti**	CHI microspheres and ALG microspheres	GEN (CHI microspheres) and VAN (ALG microspheres)	Antibiotic-loaded CHI and ALG microparticles were entrapped in porous-coated Ti to produce local drug release and inhibit adjacent bacterial growth	In Vitro	[234]
**Ti**	CHI	VAN	The coatings were biocompatible and provided an antibacterial effect, while reducing the rate of corrosion; release of VAN for up to 6 days	In Vitro	[235]
**316LVM stainless steel**	Alternating layers of CHI and the pharmaceutical	DCF	The coatings were biocompatible, provided a certain degree of corrosion protection, and improved osteointegration; controlled release of DCF	In Vitro	[18], [95]
**316LVM stainless steel**	Alternating layers of CMC and the pharmaceutical	DCF	The coatings were biocompatible, they improved osteointegration, and did not influence the corrosion susceptibility of stainless steel; controlled release of DCF	In Vitro	[17]
**316LVM stainless steel**	Alternating layers of CMC and the pharmaceutical; β-cyclodextrin for increasing the DEX dosage	DEX	The coatings were biocompatible and showed an osteointegrative potential; their application did not increase the corrosion susceptibility of stainless steel; release of DEX for up to 3 days	In Vitro	[236]
**316LVM stainless steel and** **Ti6Al4V**	Cellulose nanofibril suspension, ALG, and CMC	CLIN	The coatings were biocompatible; complete release of CLIN after 3 days	In Vitro	[237]
